# Animal magnetic sensitivity and magnetic displacement experiments

**DOI:** 10.1038/s42003-024-06269-4

**Published:** 2024-05-27

**Authors:** Kenneth J. Lohmann, Nathan F. Putman, Sönke Johnsen, Catherine M. F. Lohmann

**Affiliations:** 1 Department of Biology, University of North Carolina at Chapel Hill, Chapel Hill, NC USA; 2 LGL Ecological Research Associates, Bryan, TX USA; 3 Department of Biology, Duke University, Durham, NC USA

**Keywords:** Animal behaviour, Animal migration

**arising from** W. T. Schneider et al. *Communications Biology* 10.1038/s42003-023-04530-w (2023)

Compelling evidence that animals use magnetic maps for navigation comes from magnetic displacement experiments in which animals are exposed to magnetic signatures (combinations of magnetic intensity, inclination, and occasionally, declination) that exist in different geographic regions^1–3^. Diverse species respond to magnetic displacements as if they have been geographically displaced. In a recent paper, Schneider et al.^4^ argue that, if one assumes that sensitivity to variations in magnetic fields is low, then most magnetic signatures mark larger areas than researchers expect, and the same signatures might exist at multiple geographic locations. (Note that they use ‘sensitivity’ to mean the ability to discriminate between similar signals rather than the ability to detect a minimal signal). In their view, most magnetic signatures used previously are too ambiguous for animals to interpret. This opinion is puzzling, because, if true, then animals would presumably respond to these ‘ambiguous’ magnetic signatures with confusion and disorientation, rather than with directed movements that make sense in the context of their movement ecology, as is observed. This apparent contradiction can be resolved by recognizing that the sensitivity used by Schneider et al.^4^ deviates significantly from estimates derived empirically. Here we discuss evidence from the literature that animals may be considerably more sensitive to magnetic field parameters than analyses by Schneider et al.^4^ assume.

Schneider et al.^4^ base their analyses on what they say is “the highest likely level of sensitivity to magnetic parameters in animals”. Without providing justification, they assume that animals can detect no less than 0.5 degrees of inclination and no less than 200 nT of intensity. In reality, magnetic sensitivity has not been established definitively in any animal, but numerous studies imply considerably higher sensitivity (Tables 1 and 2).

**Table 1:** Estimates of sensitivity to magnetic inclination angle derived from empirical results

Inferred sensitivity to magnetic inclination	Reference	Method and notes
~ 0.02 degrees	Wynn et al.^5^	Changes in shearwater (*Pufffinus puffinus*) nesting locations were correlated with changes in inclination; of the 109 birds that changed location, over half moved when inclination had changed by as little as 0.01–0.02 degrees.
~ 0.04–0.05 degrees	Wynn et al.^6^	Analyses provide evidence that reed warblers (*Acrocephalus scirpaceus)* use specific (probably learned) inclination angles as a signal to terminate the return migration. See text for discussion.
~ 0.01–0.05 degrees	Rodda^15^	Changes in homing orientation of alligators (*Alligator mississippiensis)* in relation to subtle changes in Earth’s magnetic field

**Table 2:** Estimates of sensitivity to magnetic intensity derived from empirical results

Inferred sensitivity to magnetic intensity	Reference	Method and notes
<70 nT	Keeton et al.^16^	Deterioration of pigeon (*Columba livia)* homing was correlated with slight geomagnetic disturbances; see also Gould^12^
<20 nT	Dennis et al.^17^	Sensitivity inferred from an analysis of pigeon (*Columba livia)* flight paths along intensity isolines after release in a magnetic anomaly
~40 nT	Southern^18^	Disruption of orientation behavior in ring-billed gulls (*Larus delawarensis)* during slight geomagnetic disturbances
~30–60 nT	Putman et al.^9^	Changes in migratory path of sockeye salmon (*Oncorhynchus nerka*) were correlated with slight changes in Earth’s magnetic field. The entire range of intensity differences for one multi-year data set only spans ~135 nT.
~20–40 nT	Putman et al.^9^	Changes in migratory path of pink salmon (*Oncorhynchus gorbuscha*) correlated with slight changes in Earth’s magnetic field. The entire range of intensity differences for a multi-year data set only spans ~ 69 nT
~26 nT	Walker and Bitterman^19^	Best performance in honeybee (*Apis mellifera)* conditioning study
~10 nT	Kirschvink and Gould^20^	Correlation between honeybee (*Apis mellifera)* dance misdirection and slight changes in Earth’s magnetic field

For example, the assumption that 0.5 degrees of inclination represents the highest sensitivity of animals conflicts with two recent studies on birds, both of which suggest exceptional sensitivity to this magnetic parameter (Table 1). In the first, Wynn et al.^5^ demonstrate that small changes in inclination angle along a coastline are correlated with changes in nesting locations of shearwaters (*Puffinus puffinus*); of the 109 birds that changed location, the majority did so when the field changed by 0.02 degrees or less. In the second, Wynn et al.^6^ present evidence that migratory reed warblers (*Acrocephalus scirpaceus*) use specific, presumably learned, inclination angle values to mark the endpoint of their return migrations. Because recovery sites of adult birds differed on average by about 5 km from the locations predicted by the inclination model, the results are consistent with an ability to detect inclination differences of at least 0.04–0.05 degrees. Similarly, an assumption of low magnetic sensitivity is difficult to reconcile with growing evidence that some animals, such as lobsters and newts, use magnetic maps over distances of only 4–37 km^7,8^.

The assumption that 200 nT represents the limits of intensity detection in animals is also contradicted by many studies (Table 2). In two species of salmon, for example, subtle changes in field intensity have been correlated with changes in the proportion of fish that take one of two migratory routes around Vancouver Island to arrive at the Fraser River^9^. For sockeye salmon (*Oncorhynchus nerka*), every ~31 nT increase is correlated with another 10% of the salmon shifting their migratory route north^9^. For pink salmon (*Oncorhynchus gorbuscha*), every ~22 nT increase is correlated with another 10% of salmon shifting their migratory route north^9^.

Accumulating evidence thus suggests that animal magnetic sensitivity is significantly higher, and possibly even a full order of magnitude better, than Schneider et al.^4^ assume, both for inclination (Table 1) and for intensity (Table 2). This casts doubt on the validity of their analysis and, in particular, on their critiques of previously published papers, especially given that these new analyses do not change the interpretation of earlier studies^10^.

In effect, Schneider et al.^4^ reimagine the world as it might appear to an animal with poor magnetic sensitivity and a global knowledge of geography, an approach that inverts what is known and unknown. We know from magnetic displacements how an animal responds to a specific magnetic field. We do not know the animal’s magnetic sensitivity or whether the animal is aware, as Schneider et al.^4^ worry, that a similar field exists far away in a place it never goes. We do not know how large an area the signature represents to the animal (a localized area or a broad region), or whether the animal imagines itself to be anywhere at all. We do not know how sensitivity to magnetic fields is distributed within animal populations, i.e., whether population-level responses reflect the sensitivity of most individuals, or of just some particularly accurate individuals, or, instead, represent wisdom-of-the-crowd effects in which weakly sensitive individuals collectively appear to display higher sensitivity^11^. In the absence of such information, we favor a more conservative approach in which experiments reveal responses of animals, and data, rather than assumptions, shape discussion.

The emerging vision of magnetic maps is expansive, encompassing diverse strategies, the use of different magnetic parameters by different animals, and the recognition that natural selection is indifferent to whether a navigation system is perfect or fits human conceptions^1^. The precision of a map is much less important than its utility to the organism. Natural selection will favor even a rough map if it enhances survival. Indeed, some animal maps may consist entirely of simple rules that prevent animals from straying out of a favorable geographic range^1–3^.

In sum, there is general agreement that sensitivity is one factor that influences the specificity of an animal’s magnetic map^12–14^, but we find no basis for disparaging earlier work. Magnetic displacement is a robust technique for determining whether animals derive positional information from Earth’s magnetic field. We encourage our fellow researchers to eschew assumptions regarding sensitivity, focus on strong experimental design, and simply let the animals show us what they can do.
